# Attitudes of Polish Medical Students toward Organ Donation in Cases of Brain Death

**DOI:** 10.3390/ejihpe14050073

**Published:** 2024-04-23

**Authors:** Marzena Mikla, Kamila Rachubińska, Antonio Ríos, Daria Schneider-Matyka, Mariusz Panczyk, Artur Kotwas, Beata Karakiewicz, Elżbieta Grochans, Anna Maria Cybulska

**Affiliations:** 1 Faculty of Nursing, University of Murcia, 30100 Murcia, Spain; marmikla@yahoo.com; 2 Department of Nursing, Faculty of Health Sciences, Pomeranian Medical University in Szczecin, 48 Zolnierska St., 71-210 Szczecin, Poland; daria.schneider.matyka@pum.edu.pl (D.S.-M.); elzbieta.grochans@pum.edu.pl (E.G.); anna.cybulska@pum.edu.pl (A.M.C.); 3 Department of Surgery, Paediatrics, Obstetrics and Gynaecology, University of Murcia, 30100 Murcia, Spain; arzrios@um.es; 4 Department of Education and Research in Health Sciences, Faculty of Health Science, Medical University of Warsaw, 00-581 Warsaw, Poland; mariusz.panczyk@wum.edu.pl; 5 Subdepartment of Social Medicine and Public Health, Department of Social Medicine, Pomeranian Medical University in Szczecin, 71-210 Szczecin, Poland; artur.kotwas@pum.edu.pl (A.K.); beata.karakiewicz@pum.edu.pl (B.K.)

**Keywords:** brain death, knowledge, medical students, organ donation

## Abstract

(1) The aim of our study was to determine the attitudes of medical students toward organ donation in the case of brain death. (2) The study was conducted among 1348 medical students from three medical universities in Poland. The research tool was the Polish version of the standardized questionnaire concerning attitudes toward organ donation and transplantation (ODT) [PCID-DTO RIOS: A questionnaire designed by the ‘International Collaborative Organ Donation project about organ transplantation and donation]. (3) Some sources of information on organ donation were found to have a significant impact on the recipients’ knowledge of brain death. These were books, friends, family, lectures in other centers, social media, and the Church. Medical students holding the opinion that recovery and leading a normal lifestyle after brain death is impossible were significantly more likely to donate their organs after death, not for religious reasons and not because they wanted to survive their own death. (4) The medical students in our study showed a high level of awareness and favorable attitudes toward ODT. However, the number of registered donors was low. It is important to educate students on these issues to raise the awareness of both future medical professionals and the public on organ transplantation procedures. The public should be made aware that transplantation procedures are of a high standard, and that the law protects both donors and recipients. These measures would reduce recipients’ waiting time, and certainly increase the statistics of the number of life-saving and health-saving procedures.

## 1. Introduction

The development of transplantology has also contributed to the emergence of new challenges, opinions and problems. Hence, new solutions had to be found, including a new definition of death and legislation on the transplantation of cells, tissues and organs [1].

Organ transplantation is the transfer of living cells, tissues or organs within one organism or between two organisms. Brain death is defined as the irreversible cessation of the entire brain function after permanent apnea and unresponsiveness to external stimuli have been established. Modern medicine allows for the transplantation of organs, tissues and cells from living people. Organ transplantation is now a standard medical procedure, but it still raises many ethical controversies. There is a belief that basic ethical principles should be respected in the treatment process. These principles include respect for autonomy, non-maleficence, good doing, and justice [2,3].

The act on the collection, storage and transplantation of cells, tissues and organs of 1 July 2005, the so-called transplantation act is the most important legal act in Poland in the field of transplantation medicine. The act regulates and organizes the transplant system in Poland, where the legislation is consistent with European Union law, the regulations of the World Health Organization and the Council of Europe. Poland has been a member of the European Union since 2004, so the legal acts issued by it are binding on Poland. The European Union has so far adopted five directives on transplantation, three of them concern the collection, banking and transplantation of cells and tissues, and the remaining ones concern organs intended for transplantation [4]. In Poland, the state budget unit established by the Minister of Health in 1996 is the Organization and Coordination Center for Transplantation “Poltransplant”, which is responsible for supervising the organ procurement and transplantation program in Poland [4]. In Poland, according to law, brain death is confirmed by a commission consisting of three specialists—including a specialist in anesthesiology and intensive care, neurology or neurosurgery. In case of any doubt, the commission is obliged to refrain from declaring brain death. The condition for collecting organs from deceased persons is to confirm death in accordance with the regulations in force in a given country. In Poland, organs can be harvested from people with irreversible damage to the central nervous system and after brain death has been confirmed [5]. Before disconnecting the patient from any equipment, the possibility of harvesting some organs for transplantation should be considered. One of the strategic areas of hospital activity, apart from treatment, disease prevention and education, is organ donation. A hospital’s legal obligation is to report the possibility of harvesting organs from deceased persons [4]. There are only a few hours left between the moment brain death is confirmed and when organs are harvested to ascertain the deceased’s will regarding the donation of tissues and organs after death. Pursuant to the Act of 1 July 2005 on the collection, storage and transplantation of cells, tissues and organs, a doctor or a person authorized by them is obliged to determine whether the deceased person did not express any objection to the donation of organs, cells, tissues during their lifetime. This act also specifies the form of expressing opposition to the donation of organs, cells and tissues for transplantation during the lifetime of a person who is over 16 years of age. Pursuant to this act, an objection may be expressed by: entry in the Central Register of Objections to the collection of cells, tissues and organs from human cadavers, or a written declaration with a handwritten signature, an oral declaration made in the presence of at least two witnesses, confirmed by them in writing [6]. It should be emphasized that the expressed objection is revocable and may be withdrawn in any of the three forms appropriate for its submission [7]. The Transplantation Act also applies to a situation in which the deceased person did not express any objection during their lifetime. According to this, a transplant of cells, tissues and organs can be performed from a deceased person who did not express any opposition during their life, and this is so-called presumed consent [8,9]. In Poland, despite the applicable presumed consent, doctors always ask relatives for their opinion on the removal of organs from the deceased [10]. People who wish to express their willingness to donate cells, tissues and organs for transplantation after death have the opportunity to complete a declaration of will, which is for information purposes only, and they are not obliged to report it anywhere. It has no legal value, but it is a huge expression of support for organ transplantation [8]. Statistical data provided by Poltransplant show that in 2016, 677 potential deceased organ donors were reported. Organs were harvested from 80% of those reported, while 20% of the deceased were not harvested due to lack of authorization for collection (10.8%) and for medical reasons (9%). On 31 December 2016, 29,545 declarations were registered in the Central Register of Oppositions, including 29,249 oppositions and 296 withdrawals of oppositions [10].

The development of transplantology has allowed for the treatment of people with end-stage organ failure without harm to the donor. However, the process of consenting to a transplant still encounters many difficulties. One of which is that despite the presumed consent to organ donation provided in the law, the family of the deceased should be asked for such consent. It is hardly surprising that the family of the deceased, in shock and mourning after the loss of their loved one, does not consent to organ donation. It seems that educating the public more widely about the possibilities and effects of transplantation may increase the number of donations. Also, the number of transplants from living donors, which in Poland is very low, is probably the result of low public awareness in this area [11].

Some people have negative attitudes toward brain death donation on the grounds of their religious beliefs. These beliefs probably play an important role in the decision to donate organs of the deceased for transplantation [11]. A UK report found no significant relationship between religion and attitudes toward organ donation [12], while another Pakistani study established that religious barriers were the most common reason for refusing organ donation [13]. It is, therefore, important to educate the public and provide support for people when completing an application for organ donation at the time of a loved one’s death [14].

Health care providers play a special role in enhancing public awareness of brain death and organ donation [15]. It can be assumed that healthcare professionals are the most aware of the value of organ donation. They can also influence the willingness of the general population to donate organs after death or sign up for an organ donor card.

The availability of organ donors can be improved by raising awareness and dispelling misconceptions about organ donation among the general public. A review of the literature indicates that there are few studies assessing the knowledge and awareness of brain death among medical students. According to Rios et al. [16], 67% of medical students know the concept of brain death, and this knowledge increases with the progress of their studies. Alnajjar et al. [17] reported that the respondents in their study had sufficient knowledge of organ donation and transplantation (ODT). In contrast, Ozturk Kaygusuz et al. [18] observed that most students did not know the principles of brain death. In a study by Tawil et al. [19], the level of understanding of brain death among students of a four-year accredited American medical school was low. This knowledge gap persists among graduate students because most of them do not receive sufficient training on the subject of brain death. Also, Hamano et al. [20] reported that the understanding of organ transplantation among fifth-year medical students in Japan was not outstandingly high. 

The decision to donate your organs is both important and difficult. Those who play a key role here are healthcare providers, whose knowledge and attitudes can facilitate the organ donation process. Since medical students are future healthcare workers, it is important to know both their attitudes toward ODT and the level of their knowledge of these issues. Due to the fact that available studies show different results, and there are a lot of gaps in the literature about the attitudes toward organ donation in Polish samples, we decided to carry out some research among Polish students. Therefore, the aim of our study was to determine the attitudes of medical students toward organ donation in the event of brain death.

## 2. Materials and Methods

### 2.1. Settings and Design

This was a sociological, multicenter, observational study carried out in Poland. The study was conducted among 1530 medical students from three medical universities in Poland: the Pomeranian Medical University in Szczecin, Poznań University of Medical Sciences, and the Medical University of Gdańsk. The inclusion criteria were: age over 18, medical student status at one of the three medical universities in Poland (the Pomeranian Medical University in Szczecin, Poznań University of Medical Sciences, and the Medical University of Gdańsk), and informed written consent to participate in the study. Out of 1530 medical students, 1348 met all of the inclusion criteria and correctly completed the questionnaire (completion rate: 88.10%). The rest of the students simply did not agree to participate or did not complete the questionnaire.

The study was conducted in accordance with the Declaration of Helsinki after obtaining the consent of the Bioethics Committee (KB-0012/219/06/16) and the Deans of the Faculty of Medicine at the three medical universities, namely the Pomeranian Medical University in Szczecin, Poznań University of Medical Sciences, and the Medical University of Gdańsk. To prevent selection bias, the questionnaire was used for each year of study. All of the participants were informed about the purpose of the study and the possibility of resignation at any stage. Before the questionnaire was made available to students, its structure and content were explained to them, including instructions on how to answer the questions. Participation in the study was voluntary and anonymous.

### 2.2. Research Instruments

The research tool used in the study was the Polish version of the standardized questionnaire concerning ODT (university students), consisting of 45 close-ended questions. The Polish version was developed on the basis of a validated questionnaire of attitudes toward ODT (PCID-DTO RIOS: A questionnaire designed by the International Collaborative Organ Donation project about organ transplantation and donation (Proyecto Colaborativo Internacional Donante sobre la Donación y Transplante de Organos in Spain) developed by Dr. Ríos) [16,21]. The dependent variable used in the study was attitude toward organ donation, and independent variables were: (1) marital/social status, (2) the knowledge and sources of information concerning ODT, (3) social interaction, (4) prosocial behavior, (5) religion, and (6) attitude toward the body. Cronbach’s alpha reliability coefficient for each of the subscales was: α = 0.72; α = 0.91; α = 0.92; α = 0.89, respectively.

### 2.3. Statistical Analysis

Quantitative and categorical variables were described using descriptive statistics methods. The measures determined for quantitative variables were central tendency (mean, M) and dispersion (standard deviation, SD). The measures determined for categorical variables were: number (N) and frequency (%). The socio-demographic data were presented as the number of cases and %, and the group equipotency was verified with the chi-square test (χ^2^).

All of the calculations were performed using Statistica 13.3 (TIBCO Software, Palo Alto, CA, USA). For all analyses, *p* < 0.05 was considered statistically significant.

### 2.4. Brief Characteristics of the Respondents

Of the 1530 medical students invited to complete the survey, 1348 correctly completed and returned the questionnaires. The study sample included 525 (38.9%) men and 823 women (61.1%). The median age was 22.07 years (SD ± 2.13 years).

## 3. Results

### 3.1. Opinion on Clinical Death

We analyzed the influence of sociodemographic (age, sex, place of residence) and university (university, year of study) variables on the students’ opinions about brain death.

Our analysis demonstrated that sex, year of study, and place of study had a statistically significant impact on the opinions about the possibility of recovery and leading a normal life after being declared brain dead.

Most of the respondents claimed that it is impossible to recover and lead a normal life after brain death, with more women than men holding this opinion (*p* = 0.004). The students from Poznań University of Medical Sciences were more likely to believe that brain death prevents recovery and normal functioning than students from other medical universities (*p* = 0.015). These opinions also depended on the year of study (*p* = 0.001).

Other variables (age and place of residence) did not have a statistically significant effect on the knowledge concerning brain death (*p* > 0.05) (Table 1).

We analyzed the sources from which the medical students obtained information about brain death and the possibility of organ donation (Table 2).

There were statistically significant differences between the students in terms of their opinions about the possibility of recovery and leading a normal life for brain-dead individuals, depending on the sources of information. These differences were observed for books (*p* = 0.007), friends (*p* = 0.002), family (*p* = 0.006), lectures in other centers (*p* = 0.000), social media (*p* = 0.001), and religious sources (*p* = 0.005). Other sources had no impact on the opinions of medical students on brain death (Table 2).

### 3.2. Organ Donation after Death and the Knowledge of Brain Death

We analyzed the relationship between the knowledge of brain death and the decision to donate organs after death.

Medical students holding the opinion that recovery and leading a normal lifestyle after brain death is impossible were significantly more likely to donate their organs after death, not for religious reasons (*p* = 0.002) and not because they wanted to survive their own death (*p* = 0.003) (Table 3).

Another aspect analyzed in our study was the relationship between the students’ opinions on the possibility of recovery and leading a normal life after being declared brain dead and such aspects as being a blood donor, the fear of desecration of the body after death, the opinions of loved ones (father, mother, partner) about ODT, and the need to be an organ recipient in the future. The data are shown in Table 4.

Based on the data, it was found that the majority of medical students who know that one cannot recover after brain death are not afraid that after death their bodies will be desecrated (*p* = 0.000) and believe that they can be organ recipients in the future (*p* = 0.015).

Among those who believe that recovering after brain death is impossible, the majority did not know their father’s opinion about ODT (*p* = 0.043), while they knew that their mother (*p* = 0.000) and life partner (*p* = 0.001) had a positive opinion about ODT.

No statistically significant relationship between blood donation and the knowledge of clinical death was observed in the study (Table 4).

We analyzed the relationship between the students’ religion, the opinion of their church on organ donation, a law that would allow state institutions to take the organs of the deceased without prior permission, and the knowledge of brain death.

Based on the data, the majority of those aware that it is impossible to recover and function normally after brain death were practicing Catholics (*p* = 0.000) and those convinced that their church was against organ donation after death (*p* = 0.022). Moreover, among those who considered brain death as the end of life, the majority claimed that there should be a law that allows state institutions to take organs of the deceased without prior permission (*p* = 0.022) (Table 5).

## 4. Discussion

Our study of medical students demonstrated that their opinions on the possibility of recovery and leading a normal life for brain-dead people were statistically significantly related to their sex, year of study, and place of study. The majority of them believed that it is impossible to recover and lead a normal life after brain death, with more women than men holding this opinion. Students from Poznań University of Medical Sciences were more likely to assert that brain death prevents recovery and normal functioning than students from other medical universities. Also, the year of study had a significant impact on the students’ opinions about brain death. This may indicate that with years of experience and knowledge during study, they become aware that there is no possibility of recovery after the diagnosis of brain death.

According to Kolagari et al. [22], sex is significantly related to the level of knowledge and adopted attitudes. These authors found that women had greater knowledge and more positive attitudes toward ODT than men. Their findings are consistent with those reported by Almutairi et al. [23], and contrary to the results obtained by Wolide et al. [24]. This may be because women are often more empathetic, sensitive and caring. Thus, the emotional quotient may play a greater role in women’s responses, especially in the event of adversity or crisis. It is commonly believed that women are more willing to make sacrifices and probably respond more favorably to organ donation, especially if there is a need in their family. It is widely believed in society that men, who are the “breadwinners”, may be more reluctant to donate their organs compared to women, whose decision to donate is largely influenced by their parents, spouses or both. However, there are also studies that do not confirm the existence of any relationship between sex and the knowledge of organ donation [25,26,27,28,29,30].

We have noted that some sources of information on organ donation had a significant impact on the recipients’ knowledge of brain death. These were books, friends, family, lectures in other centers (*p* = 0.000), social media, and the Church. Other sources had no such impact. Kolagari et al. [22] and Alizadeh Taghiabad et al. [31] provided evidence that television was the most frequently used source of information on ODT. This is in line with the reports of other scientists [32,33,34,35,36,37,38,39,40,41], who prove that the mass media play an essential role in encouraging people to donate organs. In a study by Zahmatkeshan et al. [42], apart from the media, the main sources of students’ information on organ donation after brain death were their professors and textbooks. As stated by Ozturk Kaygusuz et al. [18], on the other hand, students obtained most of the information on ODT from family and friends. A review of the literature indicates that the demand for information on organ transplantation decreased after university classes, while the willingness to donate organs increased, especially among fourth-year students [43].

We observed in our study that medical students who were convinced that it is impossible to recover and live a normal life after brain death were far more likely to decide to donate their organs after death, not for religious reasons and not because they wanted to survive their own death. Alnajjar et al. [17] found that the majority of medical students understood the concept of brain death, but unfortunately, some of them were not sure that brain death equates to the death of the patient. In contrast, Ozturk Kaygusuz et al. [18] observed that the majority of students did not know the principles of brain death. In a study by Tawil et al. [19], the level of understanding of brain death among students of a four-year accredited American medical school was low. This knowledge gap persists among graduate students because most of them do not receive sufficient training in the subject of brain death. Also, Hamano et al. [20] reported that the understanding of organ transplantation among fifth-year medical students in Japan was not outstandingly high.

A review of the literature shows that religious beliefs influence attitudes toward transplantation among medical students [21]. According to Burra et al. [44], medical students do not accept organ donation mainly because they doubt the relevance of the criteria adopted for brain death. Tagizadieh et al. [45], on the other hand, showed that the main reasons for students’ reluctance to become organ donors were religious beliefs and a sense of violation of the integrity of the body. Hamed et al. [46] attributed the reluctance to organ donation to a lack of trust in a highly commercialized health care system.

The influence of religion on attitudes toward organ donation is controversial [47]. A UK report found no significant relationship between religion and attitudes toward organ donation [12], while another Pakistani study established that religious barriers were the most common reason for refusing organ donation [13]. Our investigation showed that the majority of medical students who were aware that there is no recovery from brain death were not afraid that their bodies would be desecrated after death, and believed that there was a possibility that they would need organs for transplantation in the future. Respondents who claimed that one cannot recover and function normally after brain death were mostly practicing Catholics and those who believed that their church was against organ donation after death. What is more, among those who considered brain death to be the end of life, the majority held the opinion that there should be a law that allows state institutions to take organs of a deceased person without prior permission [48,49].

Michał Nowicki, who analyzed the number of transplants performed in various countries of the world, claims that religion is a factor that plays an important role in the development of transplantology [50]. However, most religions and ethnic groups do not oppose organ transplantation. The exceptions are the Roma (gypsies), as well as followers of Confucianism and Shintoism, who resist organ removal from deceased donors. In fact, religions do not persuade their followers either to donate organs or to refuse to undergo a transplant. Despite this, the number of transplants performed around the world varies greatly between countries. This is because until now, there was a belief that undergoing this procedure is against religion and tradition [21]. Educational, socioeconomic, cultural, and religious factors, as well as the knowledge of and attitudes toward organ donation influence the decision to donate organs during life and after death [51,52,53,54,55,56].

According to the Organizational and Coordination Center for Transplantation Poltransplant, in 2022, 1402 organs from deceased donors were transplanted in Poland, over 120 more than a year earlier. Last year, there was a dramatic increase in these procedures—1805 internal organs were transplanted, such as kidneys, heart, liver, pancreas and lungs. In 2012, 1546 organs were transplanted, 133 more than in 2011. In the following two years, the number of internal organ transplants exceeded 1500. In 2013, 1536 such transplants were performed, and in 2014, 1531 were performed. Then stagnation occurred. In 2015, there were 1432 transplants, in 2016, there were 1469, and in 2017, the number only temporarily increased to 1531. In 2018, another decline was recorded; only 1390 internal organs were transplanted. The situation improved in 2019, when 1475 such procedures were performed. However, the pandemic brought another breakdown: in 2020, only 1180 transplants were performed, and in 2021, 1274 were performed [57]. 

In 2020, 31 kidneys from living donors were transplanted in Poland. For comparison, in the same year, 284 transplants from living donors were performed in Italy, 558 in Great Britain, and 116 in Sweden. These data show that, compared to other countries, Poland has a relatively low rate of living donation [58]. It seems that despite the awareness and favorable attitudes toward ODT among medical students, the number of registered donors among them was low. 

### 4.1. Limitations

The considerations presented here on the attitudes of Polish medical students toward organ donation in cases of brain death indicated certain limitations. Firstly, the cross-sectional design of this study limits our ability to draw clear conclusions about cause and effect. Secondly, the results may not be generalizable to all students of medical universities in the country or abroad. The group of respondents should be expanded to include students from other medical universities. Furthermore, program differences regarding the subject of brain death should be taken into account. Despite its limitations, our study provides important findings, and may be a starting point for broader research on attitudes toward transplantation.

### 4.2. Implications for Practice

Our research has implications for professional practice. Our study shows that medical students should receive better education on brain death and organ donation, which could improve their future communication with patients’ families, and thus increase the number of transplants. It is important to implement effective methods, such as workshops to improve public awareness of issues about organ donation, so that they can make their own conscious decision to be a donor.

## 5. Conclusions

The medical students in our study showed a high level of awareness and favorable attitudes toward ODT. However, the number of registered donors was low. It is important to educate students on these issues to raise the awareness of both future medical professionals and the public on organ transplantation procedures. The public should be made aware that transplantation procedures are of a high standard, and that the law protects both donors and recipients. These measures would reduce the recipients’ waiting time and certainly increase the statistics of the number of life-saving and health-saving procedures. 

## Figures and Tables

**Table 1:** Opinions of the medical students about the possibility of recovery and leading a normal life after being declared brain dead, depending on their university and sociodemographic data.

Variable		Do You Think It Is Possible to Recover and Lead a Normal Life after Being Declared Brain Dead?							
		No (n = 1160)		Yes (n = 37)		Do Not Know (n = 138)		χ^2^	P
		n	%	n	%	n	%		
Sex	Male (n = 525)	449	38.7	24	64.9	50	36.2	10.856	0.004
	Female (n = 823)	711	61.3	13	35.1	88	63.8		
Place of residence	Rural area (n = 118)	100	8.6	4	10.8	14	10.1	1.483	0.830
	Small town (n = 242)	203	17.5	8	21.6	27	19.6		
	Big city (n = 988)	857	73.9	25	67.6	97	70.3		
Year of study	First (n = 232)	175	15.1	12	32.4	41	29.7	29.762	0.001
	Second (n = 469)	416	35.9	10	27.0	40	29.0		
	Third (n = 345)	296	25.5	9	24.3	37	26.8		
	Fourth (n = 119)	106	9.1	2	5.4	9	6.5		
	Fifth (n = 106)	93	8.0	3	8.1	8	5.8		
	Sixth (n = 78)	74	6.4	1	2.7	3	2.2		
University	Pomeranian Medical University in Szczecin (n = 363)	296	25.5	17	45.9	44	31.9	12.352	0.015
	Medical University of Gdańsk (n = 328)	282	24.3	10	27.0	35	25.4		
	Poznań University of Medical Sciences (n = 658)	582	50.2	10	27.0	59	42.8		

n—number of participants, %—percent of the study sample, Pearson’s chi-square test.

**Table 2:** The opinions of the medical students about the possibility of recovery and leading a normal life after being declared brain dead, depending on the source of information.

Please Indicate from Which Sources You Have Obtained Information about the Possibility of Donating Your Organs		Do You Think It Is Possible to Recover and Lead a Normal Life after Being Declared Brain Dead?							
		No (n = 1160)		Yes (n = 37)		Do Not Know (n = 138)		χ^2^	P
		n	%	n	%	n	%		
Books, brochures	yes, positive	835	74.22	20	58.82	95	71.43	14.242	0.007
	yes, negative	9	0.80	2	5.88	4	3.01		
	no	281	24.98	12	35.29	34	25.56		
Friends	yes, positive	781	69.98	20	55.56	87	65.91	16.540	0.002
	yes, negative	33	2.96	3	8.33	12	9.09		
	no	302	27.06	13	36.11	33	25.00		
Relatives	yes, positive	540	48.87	17	48.57	48	37.21	14.468	0.006
	yes, negative	61	5.52	2	5.71	17	13.18		
	no	504	45.61	16	45.71	64	49.61		
Lectures at other centers	yes, positive	307	28.11	10	29.41	39	30.00	28.994	0.000
	yes, negative	6	0.55	3	8.82	1	0.77		
	no	779	71.34	21	61.76	90	69.23		
Social media	yes, positive	625	56.87	17	48.57	70	54.26	18.317	0.001
	yes, negative	42	3.82	6	17.14	11	8.53		
	no	432	39.31	12	34.29	48	37.21		
Religious sources, ex. the Church	yes, positive	158	14.48	13	38.24	20	15.50	14.941	0.005
	yes, negative	115	10.54	3	8.82	11	8.53		
	no	818	74.98	18	52.94	98	75.97		

Pearson’s chi-square test.

**Table 3:** The students’ willingness and motivation to donate their organs after death in relation to their opinions on the possibility of recovery and leading a normal life after being declared brain dead.

If You Plan to Donate Your Organs after Death, for What Reasons?		Do You Think It Is Possible to Recover and Lead a Normal Life after Being Declared Brain Dead?							
		No (n = 1160)		Yes (n = 37)		Do Not Know (n = 138)		χ^2^	P
		n	%	n	%	n	%		
Because I think it is my moral duty	yes	590	50.86	14	37.84	69	50.00	2.444	0.295
	no	570	49.14	23	62.16	69	50.00		
Because of solidarity with those in need	yes	790	68.10	19	51.35	90	65.22	4.891	0.087
	no	370	31.90	18	48.65	48	34.78		
Because I want to survive my own death	yes	37	3.19	4	10.81	0	0.00	11.873	0.003
	no	1123	96.81	33	89.19	138	100.0		
For religious reasons	yes	51	4.40	6	16.22	10	7.25	12.111	0.002
	no	1109	95.60	31	83.78	128	92.75		
Because it is free	yes	78	6.72	5	13.51	11	7.97	2.728	0.256
	no	1082	93.28	32	86.49	127	92.03		
Because I expect someone else to do the same for me in the future	yes	442	38.10	15	40.54	51	36.96	0.169	0.919
	no	718	61.90	22	59.46	87	63.04		
Other reasons	yes	146	12.59	3	8.11	9	6.52	4.853	0.088
	no	1014	87.41	34	91.89	129	93.48		

Pearson’s chi-square test.

**Table 4:** Opinions of the medical students about the possibility of recovery and leading a normal life after being declared brain dead in relation to being a blood donor, fear of desecration of the body after death, the opinions of the loved ones about ODT, and the need to be an organ recipient in the future.

		Do You Think It Is Possible to Recover and Lead a Normal Life after Being Declared Brain Dead?							
		No (n = 1160)		Yes (n = 37)		Do Not Know (n = 138)		χ^2^	P
		n	%	n	%	n	%		
Do you donate blood?	Yes, on a regular basis	83	7.22	4	10.81	6	4.35	10.457	0.107
	Yes, sometimes or only once	245	21.32	6	16.22	33	23.91		
	No, but I wouldd like to	681	59.27	17	45.95	82	59.42		
	No and I am not going to	140	12.18	10	27.03	17	12.32		
Are you afraid that if you became an organ donor, your body would be desecrated and disfigured after death?	Yes, I do	111	9.61	8	21.62	30	21.74	26.395	0.000
	No, I do not care	788	68.23	19	51.35	73	52.90		
	I do not know	256	22.16	10	27.03	35	25.36		
Do you know your father’s opinion on organ donation for transplantation?	Yes, he is for	399	34.46	9	24.32	37	26.81	12.988	0.043
	Yes, he is against	45	3.89	4	10.81	5	3.62		
	I do not know	692	59.76	22	59.46	90	65.22		
	Other	22	1.90	2	5.41	6	4.35		
Do you know your mother’s opinion on organ donation for transplantation?	Yes, she is for	571	49.31	11	29.73	47	34.06	24.328	0.000
	Yes, she is against	81	6.99	7	18.92	10	7.25		
	I do not know	486	41.97	18	48.65	75	54.35		
	Other	20	1.73	1	2.70	6	4.35		
If you are in a relationship, do you know your partner’s opinion on organ donation for transplantation?	Yes, he/she is for	381	33.22	11	29.73	25	18.12	23.265	0.001
	Yes, he/she is against	27	2.35	2	5.41	5	3.62		
	I do not know	221	19.27	14	37.84	35	25.36		
	I am single	518	45.16	10	27.03	73	52.90		
Do you believe you will ever need an organ for transplantation?	Yes, it is possible that I will get sick	863	74.46	27	72.97	92	66.67	12.279	0.015
	No, I have a healthy lifestyle	50	4.31	5	13.51	6	4.35		
	I do not know	246	21.23	5	13.51	40	28.99		

Pearson’s chi-square test.

**Table 5:** The ability of brain-dead people to recover and lead normal lives according to the medical students, in relation to their religion, the opinion of their church on organ donation, and a law that would allow state institutions to take the organs of the deceased without prior permission.

If You Plan to Donate Your Organs after Death, for What Reasons?		Do You Think It Is Possible to Recover and Lead a Normal Life after Being Declared Brain Dead?							
		No (n = 1160)		Yes (n = 37)		Do Not Know (n = 138)		χ^2^	P
		n	%	n	%	n	%		
Are you…?	Agnostic, atheist	298	25.98	6	16.22	34	24.82	24.864	0.000
	Practicing Catholic	484	42.20	21	56.76	72	52.55		
	Non-practicing Catholic	343	29.90	6	16.22	29	21.17		
	Another religion	22	1.92	4	10.81	2	1.46		
What, do you think, is the attitude of the church you belong to toward ODT?	It is for	98	8.52	5	14.29	4	2.96	14.738	0.022
	It is against	548	47.65	8	22.86	64	47.41		
	I do not know	443	38.52	19	54.29	57	42.22		
What would you say about a law that would allow state institutions to take the organs of the deceased without prior permission?	A wonderful gesture of solidarity	98	8.52	5	14.29	4	2.96	14.738	0.022
	A good use of organs	548	47.65	8	22.86	64	47.41		
	The authority has exceeded its powers	443	38.52	19	54.29	57	42.22		
	For the family, it is like a slap in the face	61	5.30	3	8.57	10	7.41		

Pearson’s chi-square test.

## Data Availability

The data presented in this study are available upon request from the first author.
